# Characteristics of hospital admissions for pulmonary alveolar proteinosis: analysis of the nationwide inpatient sample (2012–2014)

**DOI:** 10.1186/s12890-022-02082-z

**Published:** 2022-09-24

**Authors:** Chongiin Kim, Rodrigo Garcia-Tome, Carolina Hurtado, Li Ding, Tisha Wang, Ching-Fei Chang

**Affiliations:** 1 Department of Internal Medicine, University of Southern California, Los Angeles, CA USA; 2 Department of Pulmonary, Critical Care and Sleep Medicine, University of Southern California, Los Angeles, CA USA; 3 Division of Endocrinology, Diabetes and Metabolism, Department of Internal Medicine, University of California Los Angeles, Los Angeles, CA USA; 4 Division of Pulmonary, Critical Care, and Sleep Medicine, Department of Medicine, UCLA David Geffen School of Medicine, Los Angeles, CA USA; 5 Department of Population and Public Health Sciences, Keck School of Medicine, Los Angeles, CA USA

**Keywords:** Pulmonary alveolar proteinosis, Nationwide inpatient sample, Rare clinical syndromes, Healthcare cost, Hospitalization

## Abstract

**Background:**

Pulmonary alveolar proteinosis (PAP) is a rare clinical syndrome involving the accumulation of lipid-rich proteinaceous material in the alveoli. There is a paucity of published studies on this condition. To better characterize the demographics, complication rates, mortality, and healthcare costs of patients hospitalized for PAP in the United States, a secondary analysis on the Hospital Cost and Utilization Project’s Nationwide Inpatient Sample (NIS) was performed on patients admitted from 2012 to 2014 with a diagnosis of pulmonary alveolar proteinosis.

**Methods:**

Using the NIS database, a secondary analysis was performed on 500 admissions with the diagnosis “pulmonary alveolar proteinosis.” The clinical variables and outcome measures extracted were: patient demographics, hospital costs, length of stay, frequency of admissions, and inpatient mortality rate.

**Results:**

Among a weighted estimate of 500 hospital admissions from 2012 to 2014, the number of PAP admissions averaged 4.7 per million. The population was predominantly male (55%) with a mean age of 41.45 (CI 38.3–44.5) from all socioeconomic levels. Inpatient mortality was calculated to be 5%, which may result from the fact that the majority of admitted patients had few or no comorbid conditions (CCI 0.72). The most common procedure performed during admission was a bronchoalveolar lavage. Mean length of stay was 6.2 days (CI 3.9–8.5) and average cost of admission was $29,932.20 (CI 13,739–46,124). Of note, 50% of these admissions were considered “elective.”

**Conclusions:**

Demographics of patients with PAP who have been hospitalized in the United States are similar to previously reported demographics from prior patient cohorts, specifically a male predominance and a mean age in the 40 s. The inpatient mortality rate of 5% we found is consistent with prior studies demonstrating good disease-specific survival rates. Notably, the cost per admission and overall annual cost associated with PAP hospitalization was calculated to be $29932.20 and $5 million respectively. This reflects the high economic cost associated with hospitalization of PAP patients, and provokes thought about ways to make treatment more cost-effective.

**Supplementary Information:**

The online version contains supplementary material available at 10.1186/s12890-022-02082-z.

## Background

Pulmonary alveolar proteinosis (PAP) is a rare clinical syndrome, initially reported and described by Rosen et al. [1] in 1958 characterized by lipid-rich proteinaceous material accumulating in the alveoli, resulting in dyspnea and cough. The overproduction or inadequate clearance of lung surfactant from pulmonary alveoli have three overarching etiologies: congenital, secondary, and mostly commonly, acquired or autoimmune PAP. Approximately 75% of cases can be diagnosed by bronchoalveolar lavage resulting in a characteristic opaque and milky appearance and the suggested diagnostic algorithm for those with compatible clinical and imaging findings on chest CT is to first pursue serum granulocyte–macrophage colony-stimulating factor (GM-CSF) antibody testing [2]. The treatment for PAP is tailored to its etiology. In the case of congenital etiologies, supportive care is the standard, whereas in secondary etiologies, such as in PAP associated with hematologic malignancies, treatment of the underlying disease is initiated [3, 4]. The majority of cases observed are autoimmune PAP, which constitutes approximately 92% of cases [5], and is associated with increased levels of GM-CSF antibodies [6, 7].

The most common treatment for autoimmune PAP is whole lung lavage. Various studies have attempted to determine its efficacy, and studies have previously estimated a 10–15% mortality rate from PAP-induced pulmonary failure [8], prior to whole lung lavage becoming standard of care. Although the exact indications for whole lung lavage have yet to be determined, recent studies and clinical reports generally suggest excellent response to this therapy [9–11], with long-term outcomes demonstrating clinical remission in > 70% of patients over the course of 7 years [12].

Given its relatively recent description, there is a paucity of epidemiologic data regarding the disorder itself. Historical estimates on incidence of PAP have ranged from 0.36 to 1.65 per million in different studies [13–15]. Most recently, based on large epidemiologic studies from the US and Japan, the prevalence of PAP is felt to be approximately 7 per million [2, 5, 15]. Unfortunately, the rarity of this disease has limited the amount of information we have surrounding the demographics of patients affected. In this study, we utilized the Nationwide Inpatient Sample (NIS) database in order to better characterize the demographic data, length of stay, hospitalization cost, morbidity and mortality associated with PAP.

To our knowledge, there have only been a handful of international large-database studies surrounding PAP. In the United States, there has only been one large-database study thus far which analyzed PAP hospitalizations utilizing a private insurance claims database (McCarthy et al. 2018). Thus, performing a similar study using data from the NIS will allow us to corroborate their findings and better characterize disease manifestation and inpatient healthcare costs.

## Methods

### Study design

This was a secondary analysis of discharges from U.S. hospitals with a principal diagnosis of PAP from January 2012 to December 2014; data was extracted from the NIS database. The NIS is a publicly available database through the Healthcare Cost and Utilization Project (HCUP), a family of databases that incorporates multiple state-level healthcare institutions in order to compile rich, all-encompassing representation of healthcare use in the United States. Of this family of databases, the NIS is the largest publicly available database that focuses solely on inpatient healthcare utilization. The NIS annually collects selected data elements from inpatient discharge records and provides a stratified sample of approximately 1000 hospitals in participating states, representing a sample of 20% of all inpatient discharges in the United States. Although it lacks clinical detail, its strength lies in its large sample size, with approximately 7 million discharge records evaluated annually [16, 17]. For this reason, it serves as an ideal method for studying rare diseases.

### Study population

This study included patients with a principal discharge diagnosis of PAP based on ICD-9-CM code 516.0 “pulmonary alveolar proteinosis [18]”, which resulted in a total of 500 admissions that were available for analysis (250 elective). Of note, this database is not able to differentiate between individual patients, and thus it is important to note that the population studied was not individual patients, but instances of admissions for “pulmonary alveolar proteinosis” which may include the same patient in multiple admissions.

### Outcomes

The primary outcome was frequency of hospital admissions and inpatient mortality rate. Secondary outcomes included differences in patient demographics, hospitalization costs and length of stay (LOS).

### Study variables and definitions

Patient characteristics are provided by the NIS and include age, sex, race/ethnicity, median yearly income in the patient’s zip code, insurance type, patient’s comorbidities (Charlson Comorbidity Index [CCI] for administrative data), hospital location (rural or urban), geographic region (Northeast, Midwest, West, or South), and hospital bed size. Associated conditions and procedures were identified by pre-specified ICD-9-CM codes (demonstrated in ICD-9 Additional file1: Table) and such conditions were stratified by organ system: cardiovascular, pulmonary, renal, neurologic and other. Weighted national estimates are presented based on the HCUP Methods [19]. Information on vital status (alive or death) at discharge, LOS, and hospitalization total charges are directly provided in the NIS for each hospitalization. Total hospital charges were converted to hospitalization costs based on a charge-to-cost ratio provided by the NIS. All costs were adjusted for inflation based on the Department of Labor’s consumer price index and presented in 2014 U.S. dollars (https://www.bls.gov/cpi/cpicalc.htm). Of note, the hospital costs represent the amount needed to produce the service, not the amount paid by payer. Unfortunately, the NIS database provides hospital costs that exclude the physician fees. However, an average value can be derived from data online; for example, a review of Medicare reimbursement for physician fees for whole lung lavage (CPT 3E1F88Z) from 2012 to 2014 was $362.87.

### Statistical analysis

We conducted survey analyses for the NIS data that incorporated strata, cluster, weight, and subpopulation to calculate variances. We then presented weighted national estimates, continuous variables by weighted mean (95% Confidence interval) and categorical variables by weighted frequencies and percentages. All analyses were performed using Stata/MP 14.0.

## Results

A total of 500 admissions involving the ICD-9 code 516.0 for “Pulmonary alveolar proteinosis” from the NIS were reviewed from 2012 to 2014, of which 50% (250 total) were elective admissions. The demographic data of the patients hospitalized is summarized in Table 1 below. Overall, the average age of hospitalized PAP patients was 41 years (CI 38.3–44.5). There was a slight preference for males over females (55% males) and 58% of hospitalized patients were categorized as Caucasian. There was a relatively even distribution across all levels of income, which is consistent with prior studies suggesting that PAP affects all socioeconomic levels.

**Table 1:** Characteristics of patients hospitalized with pulmonary alveolar proteinosis (2012–2014)

Total admissions	2012	2013	2014	Combined
	145	190	165	500 (50% elective)
Sex				
Ratio (M:F)	0.81	1.92	1.06	1.22
Male	65 (44.8%)	125 (65.8%)	85 (51.52%)	275 (55%)
Female	80 (55.2%)	65 (35.2%)	80 (48.48%)	225 (45%)
Mean age (years)	37.93 (CI 31.7–44.12)	44.31 (CI 39.3–49.3)	41.24 (CI 36.15–46.32)	41.45 (CI 38.3–44.59)
Age groups				
0–18 (years)	30 (20.7%)	10 (5.3%)	5 (3%)	45 (9%)
19–40 (years)	30 (20.7%)	55 (28.9%)	70 (42.4%)	155 (31%)
41–65 (years)	75 (51.7%)	115 (60.5%)	85 (51.6%)	275 (55%)
> 66 (years)	10 (6.9%)	10 (5.3%)	70 (42.4%)	25 (5%)
Race*				
White	85 (60.7%)	105 (63.6%)	75 (50%)	265 (58.24%)
Black	25 (17.8%)	35 (21.2%)	50 (33.3%)	110 (24.18%)
Hispanic	20 (14.3%)	5 (3.0%)	25 (16.7%)	50 (11%)
Asian/Pacific Islander	0	10 (6.1%)	0	10 (2.2%)
Other	10 (7.1%)	10 (6.1%)	0	20 (4.4%)
Hospital region				
Northeast	25 (17.4%)	45 (23.7%)	20 (12.1%)	90 (18%)
Midwest	40 (27.6%)	45 (23.7%)	20 (12.1%)	105 (21%)
South	35 (24.1%)	70 (36.8%)	75 (45.5%)	180 (36%)
West	45 (31%)	30 (15.8%)	50 (30.3%)	125 (25%)
Bed size hospital				
Small	20 (13.8%)	10 (5.2%)	20 (12.1%)	50 (10%)
Medium	30 (20.7%)	35 (18.5%)	45 (27.3%)	110 (22%)
Large	95 (65.5%)	145 (76.3%)	100 (60.6%)	340 (68%)
Median yearly income				
$1–39,999	35 (24.1%)	50 (26.3%)	50 (30.3%)	135 (27%)
$40–50,999	25 (17.2%)	40 (21.1%)	40 (24.2%)	105 (21%)
$51–65,999	30 (20.7%)	60 (31.5%)	30 (18.2%)	120 (24%)
> $60,000	55 (37.9%)	40 (21.1%)	45 (27.3%)	140 (28%)
Charlson comorbidity index				
0	75 (51.7%)	125 (65.8%)	75 (45.5%)	275 (55%)
1	45 (31%)	35 (18.4%)	50 (30.3%)	130 (26%)
2	20 (13.8%)	15 (7.9%)	20 (12.1%)	55 (11%)
3	5 (3.5%)	15 (7.9%)	20 (12.1%)	40 (8%)

*All monetary values were adjusted for inflation to 2014 USD

In-hospital mortality rate over the 3 years averaged to approximately 5.0%, with 7.9% in 2013 and 6.1% in 2014 resulting in patient death. Comorbid conditions were analyzed using the Charlson Comorbidity Index (CCI), with most patients having relatively few comorbid conditions, with a CCI of 0 (55%), averaging out to approximately 0.72 (CI 0–2.6). Throughout this interval period, 68% of the admissions were at “large hospitals,” characterized as either a rural hospital with 100 + beds, an urban non-teaching hospital with 200+ beds, or an urban teaching hospital with 500+ beds. The average cost of hospitalization was $29993.29 per admission, and average annual cost of hospitalization for a patient with PAP was $5071401.36 (Table 2). The average length of stay for an admission was approximately 6.28 days (CI 3.9–8.5). Complications and in-hospital interventions can be seen in Table 3.

**Table 2:** Hospitalization Measures and Outcomes (2012–2014)

*Insurance*				
Medicare	43 (29.6%)	60 (33.33%)	45 (29%)	148 (30.85%)
Medicaid	16 (11.1%)	15 (8.33%)	10 (6.5%)	41 (8.51%)
Private	75 (51.9%)	95 (52.78%)	95 (61.3%)	265 (55.32%)
Uninsured	11 (7.4%)	10 (5.56%)	5 (3.2%)	26 (5.32%)
Hospitalization cost (per capita)	$15998.60	$48447.16	$22360.27	$29932.29
Annual cost	$2319798.29	$9204961.24	$3689444.55	$5071401.36
Length of stay (days)	4.75 (CI 3.14–6.36)	8.5 (CI 2.86–14.13)	4.93 (CI 2.83–7.04)	6.24 (CI 3.92–8.55)
Death during hospitalization	0	15	10	25

**Table 3:** Procedures during hospitalizations (2012–2014)

	2012	2013	2014	Combined
Whole lung lavage	45	95	55	195
Bronchoalveolar lavage	75	85	80	240
Transbronchial biopsy	10	0	0	10
Bronchoscopy	0	20	35	55
Open lung biopsy	0	0	0	0

The most common procedure performed was a bronchoalveolar lavage, which consisted of 240 of the 500 procedures (48%) that were performed in hospitalized patients, followed by whole lung lavage, which comprised 195 of the 500 procedures (39%). The most common complications and interventions of these hospitalizations can be found in Table 4.

**Table 4:** Complications and interventions performed during hospitalizations (2012–2014)

Diagnoses	2012	2013	2014	Combined
Respiratory failure	30	50	40	120
Mechanical ventilation	25	40	35	100
Hemodialysis	5	5	10	20
Altered mental status	5	5	10	20
Sepsis	0	10	5	15
Acute CHF	0	5	10	15
Acute renal failure	0	10	5	15
Shock	0	5	0	5
Anoxic brain injury	0	0	0	0
Bacteremia	0	0	0	0
Acute coronary syndrome	0	0	0	0
Cardiac arrest	0	0	0	0
Acute ischemic stroke	0	0	0	0

## Discussion

Given the rarity of PAP, there are very few epidemiologic studies that have been performed on this patient population. To our knowledge, the largest independent cohort of patients examined remains a study by Inoue et al. that examined a total of 248 patients from a national registry in Japan [15]. Since then, there have been other, smaller cohorts looking at the demographics of this condition in Italy, Germany and China that have performed similar analyses of smaller groups [20–22]. From these studies, it appears that PAP affects males at a higher rate than females and has an association with smoking and possibly dust exposure.

It is difficult to draw a direct comparison between the data reported from our study and prior studies that have been performed by nature of the method of data collection. Given that these instances of hospitalization from the NIS do not represent individual patients, we are unable to use this data to determine the inherent incidence or prevalence of PAP in the United States [23]. It does, however, provide a large wealth of information regarding the nature of hospitalization in PAP patients, including demographic data surrounding hospitalized patients as well as the overall healthcare costs associated with the disease.

Based on previous cohorted studies from other countries, the median age of diagnosis of PAP patients ranged from 39 to 51 years [8, 15, 20, 21], whereas the mean age of hospitalized patients in our study was on the lower end at 41.45 years. One reason for this may be that patients who are at the beginning of their diagnosis tend to have symptoms requiring hospitalization and subsequent therapy, whereas older patients tend to have a lower likelihood of recurrences following therapy [24]. Given the description of a small number of patients with “spontaneous clinical remission” or entering a “quiescent phase” seen in some studies [25] following initial diagnosis, it is also possible that the younger age of hospitalization compared to cohorted studies is consistent with the natural progression of disease. As reported in prior epidemiologic studies, we also found a predominance of hospitalized male PAP patients as compared to female PAP patients (ratio of 1.22). Of note, however, the ratio favoring men was much smaller than other prior studies have seen, which usually range from 2 to 2.6. Instead, our finding was more consistent with the study from Germany by Bonella et al., who reported a ratio of 1.3 [21], as well as more recent US-based data [5] suggesting even less of a male predominance in this condition with a M:F ratio of 0.95.

The socioeconomic breakdown of inpatient hospitalization had a relatively uniform distribution across income ranges. As noted above, the patients were equally distributed across income classes from < $40,000, $40–50,999, $51–59,999, and > $60,000. The majority of patients that were admitted with PAP were White (58%) which is slightly less than the proportion of the U.S population that self-identified as White (72%) around that time, according to the 2010 U.S Census [26]. Instead, disproportionately represented were Black or African American patients who made up 24.18% of the population hospitalized, which is higher than the proportion of Black or African Americans noted in the U.S Census (12.6%) at that time. However, this data comes with the caveat that we did not control for confounding factors in this analysis, as detailed clinical information such as smoking history, are not provided by the NIS.

Interestingly, 50% of the admissions for PAP were elective admissions—which suggests that many patients may have been hospitalized with the purpose of a planned procedure, such as a therapeutic whole lung lavage [10, 11, 27]. In our study, we found that whole lung lavage was performed 195 times, while bronchoalveolar lavages were performed 240 times throughout the 3 years period. Although whole lung lavages are considered the definitive therapeutic intervention, only 79 institutions world-wide have been clearly identified that perform the procedure, and there is great variability in the execution of the procedure itself [11, 28, 29]. Our data suggests that bronchoalveolar lavages are performed more frequently (Table 3), possibly due to waning experience with whole lung lavages. Of note, one study in China noted that BAL was interchangeably used with WLL to treat the condition [22], and it is plausible that other less experienced centers have used this strategy as well. Unfortunately, given the limited nature of our database, we are unable to comment on the indications for which the BALs were performed.

There has been one study performed by McCarthy et al. [5] in 2018 that similarly used a large-scale insurance claims database that looked at approximately 5% of the US population and found an average of 109 patients yearly with the diagnosis of pulmonary alveolar proteinosis between 2008 and 2012 within their database. This study found the annual per patient healthcare costs was found to be $54,865 in this time period, and adjusted for inflation to 2014, comes to approximately $56571.69. This is significantly higher than the healthcare cost that we calculated from the NIS database, which was approximately $29,932. There is a multitude of explanations for this discrepancy in data; the initial being that while McCarthy’s data looked at total healthcare costs over a year, whereas the NIS data only includes the cost of a hospitalization. Not included in the $29,932 is the cost of prescription medications, outpatient visits, as well as emergency room visits, which may contribute to the large difference. Further, the McCarthy data was comprised of insurance claims for one healthcare provider, the UnitedHealth Group, whereas our data is representative of the insurance costs from all payers, ranging from private insurance groups to federally funded Medicare, which may skew the reported costs.

McCarthy’s study also found that their PAP patients had a longer hospital stay, with a mean length of stay (LOS) of 15.96 days. Based on our analysis of the NIS data, patients with a diagnosis of PAP had an average hospital stay length of 6.24 days (CI 3.9–8.5). Similarly, their data suggested a higher risk of comorbidity for PAP patients, with a CCI of 1.84, whereas our data found our hospitalized PAP patients to have an average CCI of 0.72 (0–2.6). Of note, while the McCarthy estimation of CCI is within the confidence interval of our study, the LOS that we found was not. These findings together tend to suggest that the patient population that we studied was overall less sick—with fewer comorbidities and a shorter hospitalization course. Although the studies were performed in different years and utilized separate insurance claims databases, there is a significant difference in LOS during hospitalization that should be closely examined in the future. The yearly mortality rate in patients that were hospitalized with PAP averaged 5.03% from 2012 to 2014 based on the NIS data. Although there have not been any studies to our knowledge looking at mortality rate, there have been studies looking at survival trends with PAP and have suggested a disease specific survival rate of > 80% at 5 years [30] with 70% of patients remaining free from recurrent PAP manifestations 7 years after initial therapy [12]. As referenced earlier, our data found 50% of total admissions to be elective admissions, which could explain the lower LOS and mortality rate.

Our study was limited by the nature of the database that we used. Unfortunately, as the NIS records instances of hospitalization rather than individual patient cases (i.e. one patient may have accounted for several admissions), we are unable to calculate the incidence or prevalence of disease, thus restricting our overall findings. Another significant limitation of our study was the inability to distinguish autoimmune PAP from other causes of PAP, such as secondary PAP, which has a significantly worse prognosis. Unfortunately, the ICD-9 code used for billing PAP (previously 516, now J84.01) does not differentiate between these conditions. However, because autoimmune PAP accounts for > 90% of cases with a low incidence of comorbid conditions, it can be hypothesized that our select NIS subgroup does accurately reflect the general population of PAP at large. Finally, we were unable to analyze other clinical factors of interest, such as smoking status, laboratory work, or diagnostic studies, because the diagnosis of PAP relied heavily on the accuracy of medical billing rather than confirmatory levels of GM-CSF autoantibodies.

Still, there are several benefits to utilizing this rich database, including the fact that it contains one of the largest collections of PAP hospitalizations in existence. By sheer sample size alone, the NIS database allows us to study rare conditions such as PAP and its associated treatment costs, thus allowing us to calculate the economic impact of the disease.

Based on the descriptive characteristics of this cohort, we found that our cohort of PAP admissions were associated with a shorter LOS and mortality rate than other studies have found. This information may be useful for clinicians as they consider elective admissions for complex medical procedures, such as whole lung lavage and weigh the risks and benefits of this decision in a rare condition such as PAP.

## Conclusions

Here we report on some demographic findings of patients who were hospitalized with PAP in the United States from 2012 to 2014. Using the NIS database, we found that the inpatient population was predominantly Caucasian (58%), male (55%), with a mean age of 41.45 (CI 38.3–44.5), spread equally across all socioeconomic levels. Our data helps confirm previous studies of cohorted patients that found an average age of presentation to be around the 4th decade. Mean length of stay was 6.2 days (CI 3.9–8.5), with 50% of admissions being categorized as elective. Mean length of stay for PAP patient admissions with concurrent WLL was 3.21 days (CI 1.56–4.85). Mean inpatient mortality was calculated to be 5% during this time period, perhaps due to the fact that patients were relatively healthy, and with few complicating comorbidities (CCI 0.72) and mostly admitted electively. This is also consistent with prior data suggesting a good response to clinical therapy, including whole lung lavage. Interestingly, our study demonstrated that the previous gold-standard treatment of whole lung lavage was only utilized in 39% of cases. This may be due to either waning clinical experience with the procedure, and/or improved alternative therapies such as supplementation with GM-CSF.

The average cost of hospitalization for PAP patients of $29932.29 far exceeded the mean cost of hospitalization in the United States, which was $10,900 in 2014 [31]. This may be due to the fact that ‘respiratory failure’ was listed as one of the top ten most expensive conditions to treat in 2017 [16]. Certainly, specialized interventions for PAP such as whole lung lavage and off-label use of GM-CSF supplementation (both inhaled or subcutaneous) are costly to perform. Given the obfuscated nature of billing and reimbursement in the United States healthcare system with various insurance structures in place, the true cost of interventions is difficult to elucidate. Understanding the current landscape in regards to PAP admissions is vital in furthering research in preventative and cost-effective care for these patients, especially given the disproportionate representation by African Americans, who already constitute a population at risk for health care disparity.

## Supplementary Information

**Additional file 1:** ICD-9 Table Codifying Comorbidities.

## Data Availability

Data was obtained from a publicly available database under the Hospital Cost and Utilization Project’s Nationwide Inpatient Sample, which can be accessed: https://www.hcup-us.ahrq.gov/db/nation/nis/nisdbdocumentation.jsp.

## Abbreviations

BAL: Bronchoalveolar lavage
CCI: Charlson-comorbidity index
GM-CSF: Granulocyte macrophage-colony stimulating factor
HCUP: Healthcare cost utilization project
ICD-9: International classification of diseases-9
LOS: Length of stay
NIS: Nationwide inpatient study
PAP: Pulmonary alveolar proteinosis
WLL: Whole lung lavage
