# Supplementary material for: Characteristics of hospital admissions for pulmonary alveolar proteinosis: analysis of the nationwide inpatient sample (2012–2014)
Source: BMC Pulm Med. 2022 Sep 24;22:365. doi: 10.1186/s12890-022-02082-z (PMC9509629; doi:10.1186/s12890-022-02082-z)
Supplement: Supplementary file 1 — Additional file 1: ICD-9 Table Codifying Comorbidities. [file 12890_2022_2082_MOESM1_ESM.docx]

| Variable | Description |
| --- | --- |
| Mortality | Indicates in-hospital death: (0) did not die during hospitalization, (1) died during hospitalization |
| Length of stay | Length of stays described in days counting admission day until discharge day. |
| Hospitalization total charges | Total hospitalization charged in US dollars |
| Age | Age in years coded 0-124 years. Subgroups: Age 18-40 years, Age 41-65 years, Age more than 65 years. |
| Sex | Indicates gender for NIS beginning in 1998: (0) male, (1) female |
| Race | Race, uniform coding: (1) white, (2) black, (3) Hispanic, (4) Other: Asian, Pacific Islander, Native American |
| Median yearly income | Median household income quartiles for patient's ZIP Code. For 2008, the median income quartiles are defined as: (1) $1 - $38,999; (2) $39,000 - $47,999; (3) $48,000 - 62,999; and (4) $63,000 or more. |
| Insurance | Expected primary payer, uniform: (1) Medicare, (2) Medicaid, (3) private including HMO, (4) self-pay, (5) no charge, (6) other |
| Charlson Comorbidity index | Charlson Comorbidity Index categorizes [comorbidities](http://mchp-appserv.cpe.umanitoba.ca/viewDefinition.php?definitionID=102446) of patients based on the [ICD-9-CM](http://mchp-appserv.cpe.umanitoba.ca/viewDefinition.php?definitionID=102932) diagnosis codes. Each comorbidity category has an associated weight based on the adjusted risk of mortality or resource use, and the sum of all the weights results in a single comorbidity score for a patient. The Charlson Comorbidity Index that resulted were further classified into a score of 0, a score of 1, a score of 2 and a score of 3 or more. |
| Hospital Geographic region | Region of hospital: (1) Northeast, (2) Midwest, (3) South, (4) West |
| Hospital bedsize | Bed size of hospital (STRATA): (1) small, (2) medium, (3) large |
| Associated conditions by organ systems | |
| Cardiovascular | |
| Cardiac arrest | ICD-9-CM code 427.5 (cardiac arrest) |
| Acute congestive heart failure | ICD-9-CM codes: 428.0 (congestive heart failure, unspecified), 428.20 (Unspecified systolic heart failure), 428.21 (acute systolic heart failure), 428.23 (acute on chronic systolic heart failure), 428.30 (unspecified diastolic heart failure), 428.31 (acute diastolic heart failure), 428.33 (acute on chronic diastolic heart failure), 428.40 (combined systolic and diastolic heart failure unspecified), 428.41 (combined systolic and diastolic heart failure acute), 428.43 (combined systolic and diastolic heart failure acute on chronic), 428.1 (left heart failure), 276.6 (fluid overload), 428.9 (heart failure unspecified), 402.01 (malignant HTN with heart failure) Excluded- 428.22, 32, 42- specific for chronic (Either systolic, diastolic or combined) |
| Acute coronary syndrome: myocardial infarction and acute angina | ICD-9-CM codes Acute Myocardial infarction according to the cardiac wall affected (410.xx) 410.00, 410.01, 410.02, 410.10, 410.11, 410.12, 410.20, 410.21, 410.22, 410.30, 410.31, 410.32 410.40, 410.41, 410.42, 410.50, 410.51, 410.52, 410.60, 410.61, 410.62, 410.70, 410.71, 410.72, 410.80, 410.81, 410.82, 410.90, 410.91, 410.92. Among these: 410.x0 (episode of care unspecified) 410.x1 (initial episode of care for a newly diagnosed myocardial infarction) 410.x2 (episode of care following the initial episode when the patient is admitted for further observation, evaluation or treatment for a myocardial infarction that has received initial treatment, but is still less than 8 weeks old). Excluded 414.xx due to chronic ischemic heart disease. Unstable angina 411.1 (intermediate coronary syndrome)  411.81 (acute coronary occlusion without myocardial infarction), 411.89 (other: acute coronary insufficiency, subendocardial ischemia), 413.0 (Angina decubitus), 413.1 (Prinzmetal Angina) 413.2 (other and unspecified Angina pectoris) |
| Pulmonary | |
| Acute respiratory failure | ICD-9-CM codes: 518.81 (Acute respiratory failure), 518.82 (Other pulmonary insufficiency, not elsewhere classified: acute repiratory distress, acute respiratory insufficiency, acute respiratory distress syndrome NES), 518.84 (acute on chronic respiratory failure) |
| Mechanical ventilation | Defined as patient with any procedural code (PR1-PR15) with the following ICD-9-CM codes: 96.7 (Other continuous invasive mechanical ventilation), 96.70 (Continuous invasive mechanical ventilation of unspecified duration), 96.71 (Continuous invasive mechanical ventilation for less than 96 consecutive hours), 96.72 (Continuous invasive mechanical ventilation for 96 consecutive hours or more), 96.04 (endotracheal intubation) |
| Renal | |
| Acute renal failure | ICD-9-CM codes: 584 (acute renal failure), 584.5 (With lesion of tubular necrosis, 584.6 With lesion of renal cortical necrosis), 584.7 (With lesion of renal medullary [papillary] necrosis), 584.8 (With other specified pathological lesion in kidney), 584.9 (Acute renal failure, unspecified). |
| Hemodialysis | Defined as patient with any procedural code (PR1-PR15) with the following ICD-9-CM codes: 39.95 (HD and hemofiltration) |
| Neurologic | |
| Altered mental status | ICD-9-CM codes: 780.97 (altered mental status), 780.0 (alteration on consciousness), 780.01 (Coma), 780.02 (Transient alteration of awareness), 780.03 (persistent vegetative state), 780.09 (other), 293.0 (delirium due to conditions classified elsewhere), 348.3 (Encephalopathy, not elsewhere classified), 348.30 (Encephalopathy, unspecified), 348.31 (Metabolic encephalopathy), 348.39 (Other encephalopathy), 293.1 (Subacute delirium), 293.8 (Other specified transient mental disorders due to conditions classified elsewhere), 293.81 (Psychotic disorder with delusions in conditions classified elsewhere), 293.82 (Psychotic disorder with hallucinations in conditions classified elsewhere), 293.83 (Mood disorder in conditions classified elsewhere), 293.89 (Catatonic disorder in conditions classified elsewhere), 293.9 (Unspecified transient mental disorder in conditions classified elsewhere) |
| Acute ischemic stroke | ICD-9-CM codes: 434.01 (Cerebral thrombosis with cerebral infarction), 434.11 (Cerebral embolism with cerebral infarction), 434.91 (Cerebral artery occlusion, unspecified with cerebral infarction), 433.01, 433.11, 433.21, 433.31, 433.81, 433.91, (Occlusion and stenosis of precerebral arteries with cerebral infarction), 434.01, 434.11, 434.91 (Occlusion of cerebral arteries with cerebral infarction) 435.0, 435.1, 435.2, 435.3, 435.8, 435.9 (Transient cerebral ischemia), 436 (Acute, but ill-defined, cerebrovascular disease). |
| Anoxic brain injury | ICD-9-CM codes: 348.1 (Anoxic brain damage) |
| Others | |
| Shock (except septic shock described below) | ICD-9-CM codes: 785.50 (Unspecified), 785.59 (other, hypovolemic), 785.51 (cardiogenic shock) |
| Sepsis | ICD-9-CM codes: 995.91: Sepsis, 995.92: Severe Sepsis, 785.52: Septic shock. Septicemia: 038-038.9, 022.3, 098.89, 054.5, 036.2, 020.2, 038.9 |
| Bacteremia | ICD-9-CM codes: 790.7 (Bacteremia) |
| Procedures | |
| Whole Lung Lavage | Defined as patient with any procedural code (PR1-PR15) with the following ICD-9-CM codes 339.9 |
| Bronchoalveolar Lavage | Defined as patient with any procedural code (PR1-PR15) with the following ICD-9-CM codes 332.4, 965.6 |
| Transbronchial biopsy | Defined as patient with any procedural code (PR1-PR15) with the following ICD-9-CM codes 332.7 |
| Bronchoscopy | Defined as patient with any procedural code (PR1-PR15) with the following ICD-9-CM codes 332.1, 332.2, 332.3 |
| Open biopsy of the lung | Defined as patient with any procedural code (PR1-PR15) with the following ICD-9-CM codes 332.8 |
